# *Abeliophyllum distichum* Ameliorates High-Fat Diet-Induced Obesity in C57BL/6J Mice by Upregulating the AMPK Pathway

**DOI:** 10.3390/nu12113320

**Published:** 2020-10-29

**Authors:** Ji Eom, Shalom Sara Thomas, Nak-Yun Sung, Dong-Sub Kim, Youn-Soo Cha, Kyung-Ah Kim

**Affiliations:** 1 Division of Natural Product Research, Korea Prime Pharmacy Co., Ltd., Gwangju 61473, Korea; j.eom@koreaprime.co.kr (J.E.); ny.sung@koreaprime.co.kr (N.-Y.S.); ds.kim@koreaprime.co.kr (D.-S.K.); 2 Department of Food Science and Human Nutrition, Jeonbuk National University, Jeonju 54896, Korea; shalomsarathomas@gmail.com (S.S.T.); cha8@jbnu.ac.kr (Y.-S.C.); 3 Obesity Research Center, Jeonbuk National University, Jeonju 54896, Korea; 4 Department of Food and Nutrition, Chungnam National University, Daejeon 34134, Korea

**Keywords:** anti-obesity, natural compound, lipogenesis, AMPK, miseon-namu

## Abstract

The use of natural compounds as anti-obesity agents has been gaining attention over the past few years. *Abeliophyllum distichum* Nakai is endemic to Korea. In the present study, an *A. distichum* leaf extract (AE) was analyzed for its anti-obesity effects in mice fed a high-fat diet. Seven-week-old male C57BL/6J mice were divided into five groups, namely, normal diet (ND), high-fat diet (HD), HD + Garcinia (GE300), HD + AE low dose (AE100), and HD + AE high dose (AE300). After 8 weeks of the experimental period, treatment with AE reduced body weight and ameliorated high-fat diet-induced changes in serum lipid levels. Histological analysis revealed that treatment with AE decreased lipid accumulation in the liver and brown adipose tissue. Also, AE reduced the adipocyte size in epididymal fat. The reduction in adipose tissue mass in the AE-treated groups was clearly visible in micro-computed tomography images. The expression levels of lipogenic genes, such as PPARγ, C/EBPα, ACC, and FAS, were significantly reduced in the AE300 group. The levels of p-AMPK and p-ACC were increased in the AE300 group compared to the HD group, indicating that the anti-obesity effect of AE was mediated through the AMPK pathway.

## 1. Introduction

Obesity is defined as the excess accumulation of fat in the body, which may threaten health by increasing the risk of diabetes mellitus, hypertension, and cardiovascular diseases. Several recent reports have shown that, globally, obesity is becoming more common in newly industrialized and low–middle-income countries, mainly owing to changes in dietary habits and a sedentary lifestyle [1,2]. By making lifestyle changes, such as increasing physical activity and consumption of a well-balanced diet, obesity can be managed effectively. However, in case of severe obesity accompanied by complications, managing body weight only through lifestyle modifications may be difficult and time-consuming. In addition, the chance of regaining weight within a few years remains an unexpected drawback [3]. Pharmacological approaches and bariatric surgery are efficient in reducing body weight. Nevertheless, many synthetic drugs used as anti-obesity agents possess adverse side effects and have been banned in several countries. Therefore, the use of natural compounds from plant and animal sources has been gaining attention over the years as a safe alternative. For example, green tea, *Garcinia cambogia,* and *Phaseolus vulgaris* are herbs proven to be effective as anti-obesity agents [4].

Adipose tissue, consisting of white adipose tissue (WAT) and brown adipose tissue (BAT), performs a significant role in managing energy homeostasis in the body. The WAT is further divided into two types: subcutaneous WAT (SAT), which is the fat layer beneath the skin, and visceral WAT (VAT), which surrounds the internal organs. In humans, VAT depots include mesenteric, perirenal, omental, and peritoneal VAT. In animal models, gonadal depots are also included in the VAT. BAT depots in humans are found around ribs and shoulders. While WAT acts as an energy reservoir for other organs, BAT stores lipids for cold-induced thermogenesis. As a response to the changing energy status of the body, the WAT undergoes rapid remodeling by changing the number and/or size of adipocytes. The other resident cells in the WAT, such as macrophages, also change simultaneously to cope with the alterations in the function of adipose tissue [5]. The WAT synthesizes and secretes several hormones, including adipokines, which regulate a wide range of processes such as nutritional intake, insulin sensitivity, and inflammatory reactions [6]. A chronic positive energy balance may lead to WAT dysfunction, where the adipocytes fail to expand any further, along with an altered expression of adipokines. These altered events lead to the accumulation of fat in organs other than WAT, such as muscle and liver, impairing their normal functioning and causing local and systemic inflammation and insulin resistance [7]. Since adipose tissue dysfunction is considered to be a mechanism related to most of the complications of obesity, natural compounds that can regulate adipogenesis and improve obesity-associated changes in the adipose tissue may be potential anti-obesity agents.

*Abeliophyllum distichum*, also identified as white forsythia, is a deciduous shrub found only in selective regions of Korea. Locally, it is known as miseon-namu. This plant belongs to the Oleaceae family, and only a single species has been reported to date. Generally known to be an ornamental plant, *A. distichum* has recently been studied for its various beneficial effects. The leaves of *A. distichum* have been reported to have anti-inflammatory effects [8]. Lee and Kang reported that an *A. distichum* flower extract suppressed lipopolysaccharide-induced inflammation and NF-kB activation in a macrophage cell line [9]. Recent reports show that *A. distichum* mitigates postmenopausal osteoporosis in rats and protects against reactive oxygen species-induced DNA damage in a murine skin fibroblast cell line [10]. However, the potential anti-obesity effect of *A. distichum* is yet to be elucidated. In a previous study in our lab, we found that a leaf extract of *A. distichum* decreased lipid accumulation in mature 3T3-L1 adipocytes (data not yet published).

In the present study, the ethanolic extract of *A. distichum* leaf (AE) was investigated for its possible anti-obesity effect against high-fat diet-induced obesity in C57BL/6J mice. We analyzed whether treatment with AE changed body weight, fat volume, and serum lipid parameters. Furthermore, we attempted to elucidate the extract’s plausible mechanism of action by analyzing changes in lipid metabolism-related genes and signaling pathways in the adipose tissue.

## 2. Materials and Methods

### 2.1. Animals and Diet

Six-week-old male C57BL/6J mice were purchased from DBL (Seoul, Korea) and were acclimatized for one week. The mice were then divided into five groups (*n* = 10 in each group), as follows: normal diet (ND), high-fat diet (HD), HD + Garcinia (GE300), HD + AE low dose (AE100), and HD + AE high dose (AE300). Normal and high-fat experimental diets contained 10% kcal% (D12450B, Research Diets, Inc., New Brunswick, NJ, USA) and 60% kcal% (D12492, Research Diets, Inc., New Brunswick, NJ, USA) from fat, respectively.

*A. distichum* for the study was obtained from Miseonnamu Products Co., (Goesan-gun, Chungcheongbuk-do, Korea). A garcinia extract (GE, 300 mg/kg body weight (b.w)) was used as a positive control. AE at doses of 100 and 300 mg/kg b.w (low dose and high dose, respectively) was administered to the mice. The samples were dissolved in 1 × phosphate-buffered saline (PBS) and orally administered for 8 weeks, once daily. The control mice were administered the same volume of 1 × PBS. The animals were sustained in a controlled environment of 23 ± 2 °C with an alternate 12 h dark and light cycle. All the experimental procedures were carried out according to the ethical guidelines of the Animal Experimental Ethics Committee of the Gyeonggi Bio Center at the Gyeonggi Institute of Economic Science and Technology (GBSA 2019-11-002). Body weight was measured weekly, and feed intake was estimated every two days.

The mice were euthanized after the experimental period to collect blood, liver, epididymal white adipose tissue (eWAT), and interscapular BAT. The serum was separated by keeping the blood at room temperature for an hour and then centrifuging at 3000 rpm for 10 min at 4 °C and was stored at −72 °C until analysis. Tissues were rinsed in PBS, weighed, frozen, and stored at −72 °C until further analysis. Tissue portions for histology were fixed in formalin.

### 2.2. Analysis of Serum Biochemical Markers

Serum lipid levels, i.e., total cholesterol (TC), triglyceride (TG), high-density lipoprotein cholesterol (HDL-C), low-density lipoprotein-cholesterol (LDL-C), were measured. Also, serum glucose (GLU) and liver injury markers such as alanine aminotransferase (ALT) and aspartate aminotransferase (AST) were measured using specific strips in a blood biochemical analyzer (BS220, Mindray, Shenzhen, China). Leptin levels were analyzed using a commercially available kit (Abcam, Cambridge, UK).

### 2.3. Micro-Computed Tomography (Micro-CT)

Micro-CT was conducted using a Quantum GX micro-CT imaging system (PerkinElmer, Hopkinton, MA, USA) at the Korea Basic Science Institute (Gwangju, Korea), to assess VAT and SAT volumes. The X-ray source was set to levels of 90 kV and 80 A with a field of view of 45 mm (voxel size, 90 μm; scanning time, 4 min). The animals received a light anesthesia (2% isoflurane/O2 gas) to immobilize them during scanning. CT imaging was represented using a 3D Viewer software within the Quantum GX. Scanning was followed by image segmentation using the Analyze software 12.0 (AnalyzeDirect, Overland Park, KS, USA). Semi-automatic and manual tools, such as object extraction, region growing, and objector separator in the Volume Editor tool were used for the segmentation analysis. Subsequently, volume measurements were calculated using the ROI tool.

### 2.4. Histological Analysis

After fixing in 10% formalin for 48 h, the tissues (liver, eWAT, and BAT) were embedded in paraffin blocks, cut into 5 µm thick sections, and stained using hematoxylin and eosin (H&E). Briefly, the sections were rinsed in xylene, dehydrated with 100, 95, 90, 80, and 70% alcohol, and stained with H&E; images were obtained using an optical microscope (Olympus, Tokyo, Japan). Image-J software (U. S. National Institutes of Health, Bethesda, MD, USA) was used to analyze the area of the adipocytes.

### 2.5. RT-PCR and Western Blot

The expression levels of genes involved in lipid metabolism in the adipose tissue, such as peroxisome proliferator-activated receptor gamma (PPARγ), CCAAT/enhancer binding protein alpha (C/EBPα), sterol regulatory element-binding protein-1c (SREBP-1c), acetyl-CoA carboxylase (ACC), and fatty acid synthase (FAS), were analyzed using RT-PCR. Briefly, mRNA was isolated from epididymal fat using the Qiagen RNeasy kit (QIAGEN GmbH, Hilden, Germany) and then reverse-transcribed to cDNA using the RT-PCR kit (TOYOBO, Osaka, Japan); the obtained cDNA was used for qRT-PCR (Bio Rad, Hercules, CA, USA) using a SYBR green qPCR mix (TOYOBO, Osaka, Japan). The primers (Bioneer, Daejeon, Korea) used in this study are listed in Table 1.

The protein levels of phosphorylated ACC (p-ACC), ACC, phosphorylated adenosine monophosphate (AMP)-activated protein kinase (p-AMPK), AMPK, and β-actin in epididymal fat were measured using western blot. Briefly, proteins extracted using RIPA buffer containing 1% phosphatase inhibitor and a protease inhibitor cocktail, were quantified, matched to the same concentration, and mixed with 5 × protein buffer. After electrophoresis on an 8–10% SDS-polyacrylamide gel, the proteins were transferred to a polyvinylidene difluoride membrane for blotting.

### 2.6. Statistical Analysis

Data are expressed as mean ± standard error of the mean (SEM). The statistical significance was verified using Duncan’s test of one-way ANOVA, SPSS version 17.0 (SPSS Inc., Chicago, IL, USA), and significance was considered at *p* < 0.05. Values with different superscript letters (a, b, c) indicate statistical significance among groups.

## 3. Results

The present study examined the effect of AE on high-fat diet-induced obesity in C57BL/6J mice. The mice were divided into five groups and fed ND or HD. The three treatment groups were orally administered GE300, AE100, or AE300 along with HD, and the control mice were given the same volume of PBS for 8 weeks along with ND or HD.

### 3.1. AE Consumption Improved High-Fat Diet-Induced Body Weight Gain

**Figure 1:**
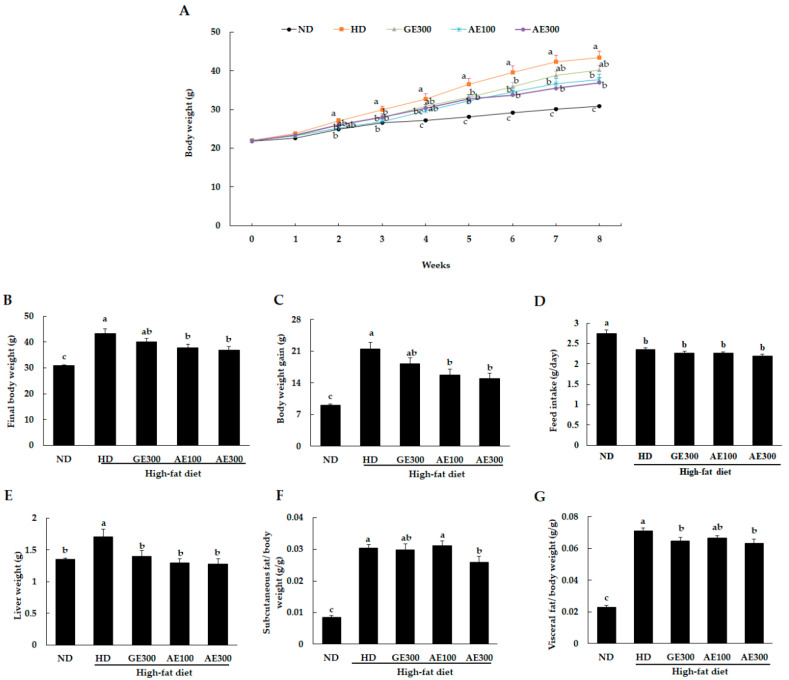
Effect of *Abeliophyllum distichum* extract (AE) on body weight and liver and fat weight in the experimental mice. Mice were fed a high-fat diet and orally administered a Garcinia extract (300 mg/kg b.w) or an *A. distichum* leaf extract at a low (100 mg/kg b.w) or at a high dose (300 mg/kg b.w) for 8 weeks. (**A**) Body weight during the experimental period, (**B**) final body weight, (**C**) body weight gain, (**D**) feed intake, (**E**) liver weight, (**F**) subcutaneous fat weight, and (**G**) visceral fat weight. ND, normal diet, HD, high-fat diet, GE300, high-fat diet + Garcinia 300 mg/kg b.w, AE100, high-fat diet + 100 mg/kg b.w, AE300, high-fat diet + 300 mg/kg b.w. Values are expressed as mean ± SEM, *n* = 10. The varying superscript letters indicate a statistically significant difference among the groups by Duncan’s test of ANOVA, *p* < 0.05.

As shown in Figure 1A,B, all the HD-fed groups had significantly higher body weights compared to the ND group. Treatment with AE significantly reduced body weight gain compared to HD alone. The HD group showed a significantly higher body weight than the ND group after the two weeks of the experimental period. There were significant differences in the body weight of the treated groups compared to those of the HD group starting from week 5. The effect of AE on body weight gain was more prominent than that of GE (Figure 1C). Though feed intake between ND- and HD-fed groups was significantly different, there was no significant difference among the HD-fed groups (Figure 1D). The HD-induced increase in liver weight was significantly reversed by treatment with GE and AE (Figure 1E). As shown in Figure 1F,G, the ratios of subcutaneous and visceral fats to body weight were significantly reduced in mice fed AE300 compared to those fed only HD. GE300 significantly reduced the visceral fat-to-body weight ratio and showed a tendency to reduce subcutaneous fat.

### 3.2. AE Improved Serum Lipid Profile, Serum Glucose Levels, and Hepatotoxicity Markers

As shown in Table 2, the levels of serum TC, TG, and LDL-C were increased in the HD-fed groups compared to the ND group. Treatment with GE and AE significantly reversed these changes in the serum lipid profile. Furthermore, GE and AE reduced the levels of the high-fat diet-induced increase in serum glucose levels. The diagnostic markers of hepatotoxicity, including ALT and AST, were significantly reduced in the GE and AE groups. Leptin levels were increased in all HD-fed groups compared to the ND group. Compared to the HD group, leptin levels were significantly lower only in the AE300 group among all the treated groups.

### 3.3. AE Reduced Subcutaneous and Visceral Fat Volumes and Decreased Ectopic Lipid Accumulation

**Figure 2:**
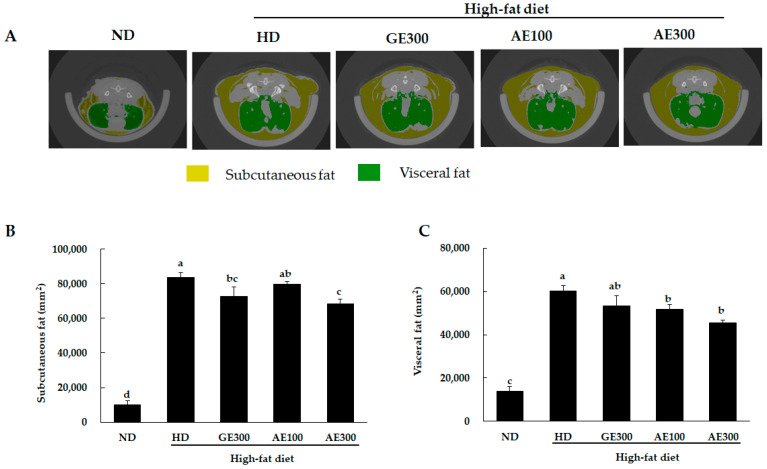
Effect of AE on abdominal fat volume in the experimental mice. Mice were fed a high-fat diet and orally administered a GE (300 mg/kg b.w) or an AE (leaf extract) at low dose (100 mg/kg b.w) or high dose (300 mg/kg b.w) for 8 weeks. (**A**) Micro-CT image of the abdomen region, (**B**) subcutaneous fat volume, and (**C**) visceral fat volume. ND, normal diet, HD, high-fat diet, GE300, high-fat diet + Garcinia 300 mg/kg b.w, AE100, high-fat diet + 100 mg/kg b.w, AE300, high-fat diet + 300 mg/kg b.w. Values are expressed as mean ± SEM, *n* = 5. The varying superscript letters indicate a statistically significant difference among the groups by Duncan’s test of ANOVA, *p* < 0.05.

**Figure 3:**
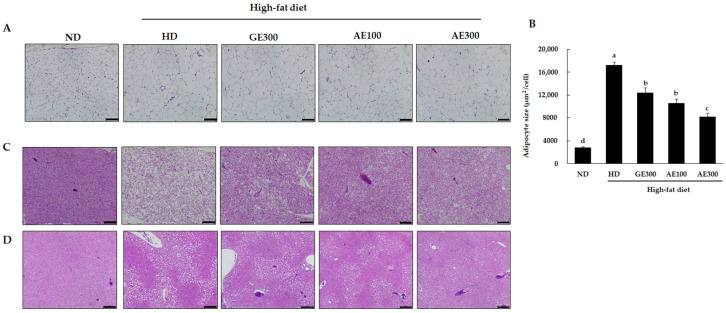
Effect of AE on epididymal white adipose tissue (eWAT), brown adipose tissue (BAT), and liver in the experimental mice, observed by H&E staining. Mice were fed as previously indicated. (**A**) eWAT, (**B**) adipocyte size, (**C**) BAT, and (**D**) liver. Scale bar = 200 µm. ND, normal diet, HD, high-fat diet, GE300, high-fat diet + Garcinia 300 mg/kg b.w, AE100, high-fat diet + 100 mg/kg b.w, AE300, high-fat diet + 300 mg/kg b.w. Values are expressed as mean ± SEM, *n* = 10. The varying superscript letters indicate a statistically significant difference among the groups by Duncan’s test of ANOVA, *p* < 0.05.

The images obtained from the micro-CT analysis revealed that treatment with a high dose of AE reduced the subcutaneous and visceral fat volumes compared to administration of HD (Figure 2A). SAT and VAT volumes were significantly decreased in the AE300 group compared to the HD group (Figure 2B,C). Furthermore, H&E staining of epididymal WAT showed that the size of adipocytes was significantly increased in the HD group compared to the ND one, as shown in Figure 3A,B. However, supplementation with GE, AE100, and AE300 significantly reduced adipocyte size. H&E staining of brown adipose tissue (Figure 3C) and liver (Figure 3D) revealed that HD-induced ectopic lipid accumulation was significantly reversed by treatment with GE300, AE100, and AE300. Interestingly, this reversal effect was more prominent in the AE300 group.

### 3.4. AE Suppressed the mRNA Expression of Lipogenic Genes in Epididymal Fat Tissue

**Figure 4:**
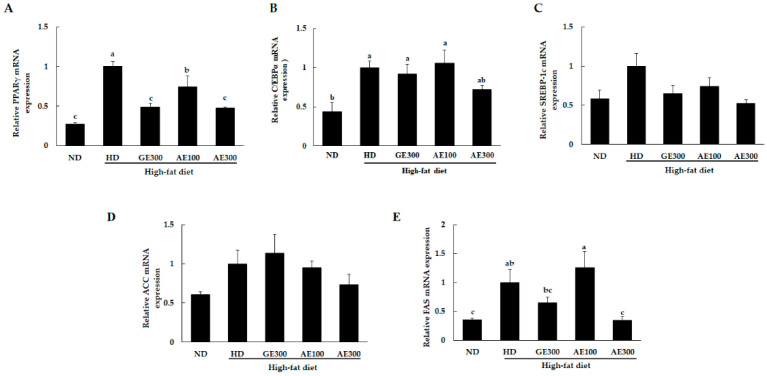
Effect of AE on the mRNA expression of genes involved in lipogenesis in the adipose tissue of the experimental mice. Mice were fed as previously indicated. (**A**) PPARγ, (**B**) C/EBPα, (**C**) SREBP-1c, (**D**) ACC, and (**E**) FAS. ND, normal diet, HD, high-fat diet, GE300, high-fat diet + Garcinia 300 mg/kg b.w, AE100, high-fat diet + 100 mg/kg b.w, AE300, high-fat diet + 300 mg/kg b.w. Values are expressed as mean ± SEM, *n* = 5. The varying superscript letters indicate a statistically significant difference among the groups by Duncan’s test of ANOVA, *p* < 0.05.

From the metric and biochemical parameters, it was evident that AE improved high-fat diet-induced obesity. To elucidate the mechanism by which AE exerts its anti-obesity effect, changes in the expression of genes related to lipid metabolism in the adipose tissue were analyzed using RT-PCR and western blotting. As shown in Figure 4A,B, the expression of adipogenic transcription factors, such as PPARγ and C/EBPα, were significantly increased in the HD groups compared to the ND group. Treatment with GE300 and AE300 reduced the levels of PPARγ, while treatment with AE300 showed a tendency to reduce the levels of C/EBPα. Furthermore, the mRNA expression levels of SREBP-1c (*p* = 0.059) and ACC (*p* = 0.137) showed a decreasing tendency in all the treated groups, while FAS was significantly lowered in the AE300 group.

### 3.5. AE Upregulated AMPK Activation in Epididymal Fat Tissue

**Figure 5:**
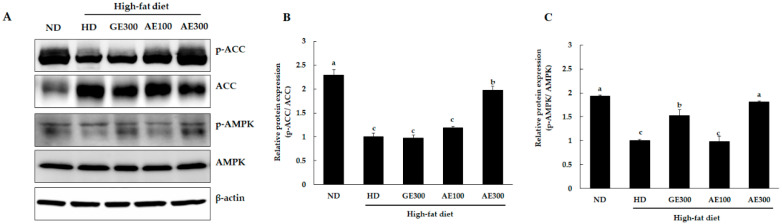
Effect of AE on proteins involved in lipid metabolism in the adipose tissue of the experimental mice. Mice were fed as previously indicated. ACC, acetyl-CoA carboxylase, p-ACC, phosphorylated ACC, AMPK, adenosine monophosphate (AMP)-activated protein kinase, p-AMPK, phosphorylated AMPK. (**A**) Western blot, (**B**) relative expression of p-ACC, and (**C**) relative expression of p-AMPK. Values are expressed as mean ± SEM of four separate experiments. ND, normal diet, HD, high-fat diet, GE300, high-fat diet + Garcinia 300 mg/kg b.w, AE100, high-fat diet + 100 mg/kg b.w, AE300, high-fat diet + 300 mg/kg b.w. The varying superscript letters indicate a statistically significant difference among the groups by Duncan’s test of ANOVA, *p* < 0.05.

As shown in Figure 5, the results of western blot analysis revealed that AE300 and GE300 significantly increased the levels of p-AMPK compared to control HD. Simultaneously, the levels of p-ACC increased significantly in the AE300 group compared to the HD group. These data suggested that AE exerts its anti-obesity effect by upregulating the AMPK pathway, which in turn suppresses the action of lipogenic enzymes such as ACC by phosphorylation.

## 4. Discussion

Obesity is characterized by increased fat mass and body weight. At the biochemical level, obesity results in an abnormal increase in the circulating levels of various molecules, such as lipids, glucose, and free fatty acids, which may pose a potential threat to the normal functioning of blood vessels and organs. In short, obesity is a significant risk factor for the development of conditions such as hypertension, insulin resistance, diabetes mellitus, and cardiovascular diseases [11,12]. Behavioral changes, including a well-balanced diet and increased physical activity, remain the first step towards reducing body weight. However, pharmacological interventions along with behavioral changes may reduce body weight more effectively [13]. The development of pharmacological therapies and nutraceuticals from natural compounds has gained attention in recent years due to the relative safety of these treatments compared to synthetic drugs [14].

In the present study, we investigated the effect of *A. distichum*, a plant endemic to Korea, against HD-induced obesity in mice. AE was orally administered to mice along with a high-fat diet for 8 weeks. The results indicated that treatment with AE significantly reversed obesity-induced changes in serum lipids and reduced hepatotoxicity markers. Furthermore, the expression of lipogenic genes in the adipose tissue was significantly suppressed by AE treatment.

Prolonged consumption of HD results in increased body weight and fat mass [15]. In the present study, the HD group showed a significantly higher body weight than the ND group, indicating that obesity was successfully induced. Treatment with AE showed a significant reduction in the final body weight and body weight gain compared to HD, irrespective of the dose. Furthermore, there was no significant difference in feed intake between the HD group and the treatment groups, indicating that the reduction in body weight was due to the effect of the AE supplementation. Interestingly, the ratios of subcutaneous and visceral fat deposits to body weight were significantly reduced only in the AE300 group. The results of the micro-CT image analysis were also in agreement with these data. The effect of AE on fat volume was stronger when compared to that of the same concentration of GE. Chronic obesity leads to the accumulation of fat in tissues, such as the liver, muscle, and heart, which occurs as a result of the failure of the adipose tissue to handle the increased energy influx [16]. AE significantly reversed the high-fat diet-induced ectopic lipid accumulation in the liver, as evidenced by the reduced liver weight/body weight ratio and the reduced number of globules in the H&E-stained images of liver tissues. In addition, the levels of serum markers of liver injury, including ALT and AST, were noticeably reduced by AE treatment compared to HD alone. In summary, these results suggest that AE may improve the visible features of obesity, and this effect is similar to that of GE.

Increased visceral fat in obesity is associated with dyslipidemia, which is a term collectively used to describe increased TG, decreased HDL-C, and increased LDL-C. Dyslipidemia increases the risk of coronary artery disease (CAD) [17]. The results of the present study show that AE improved the changes in serum lipids by decreasing TG and LDL-C levels and simultaneously reducing TC levels. Obesity is a major risk factor for the development of hyperglycemia associated with type 2 diabetes. This is the result of increased glucose output by the liver through gluconeogenesis and glycogenolysis and reduced glucose uptake by the muscles [18]. AE treatment lowered the HD-induced increase in serum glucose levels. Leptin is a major hormone that is produced by the adipocytes, which regulates hunger and satiety. High concentrations of leptin are found in the serum of obese patients and are associated with an increased risk of cardiovascular and other metabolic diseases [19]. In our study, leptin levels were significantly reduced in the high-dose AE group, while the GE- and low-dose AE- treated groups showed a tendency to reduce them. Altogether, these data indicate that AE may not only reduce body weight but also ameliorate obesity-associated complications.

WAT is involved in several physiological functions. Adipose tissue has been considered only as an energy reservoir for a long time; however, it also plays the role of an endocrine organ, which synthesizes various bioactive chemicals that synchronize metabolic homeostasis [6]. Under normal conditions, when there is a positive energy balance, adipocytes secrete leptin that reduces hunger and increases energy expenditure. In obesity, where there is chronic exposure to a positive energy balance, WAT fails to expand further to accommodate excess fat and becomes dysfunctional. A series of metabolic changes accompany adipocyte dysfunction. For instance, excess fat is deposited in other tissues that modulate glucose homeostasis, which may result in insulin resistance and increase the risk of hyperglycemia [7]. Furthermore, dysfunction of WAT also causes an imbalance in the production of adipokines and activates inflammatory pathways that contribute to the development of diseases associated with obesity [20]. In the present study, AE improved body weight and reversed the high-fat diet-induced changes in serum lipids, leptin, and glucose levels. These results collectively suggest that AE exerts an anti-obesity effect. To explain its mechanism of action, we analyzed the expression of genes associated with lipogenesis in the adipose tissue.

Lipogenesis is the synthesis of fatty acids that occurs mainly in the adipose tissue and also in the liver, depending on the diet [6]. PPARγ is a major transcriptional factor that modulates both glucose and lipid metabolism. It is expressed mainly in WAT and BAT. PPARγ expression in the adipose tissue is associated with HD-induced adipocyte hypertrophy and insulin resistance [21,22]. In addition, PPARγ and C/EBPα are crucial regulators of adipogenesis [23]. SREBP-1c is the transcription factor involved in lipogenesis and is activated by insulin [24]. ACC catalyzes the synthesis of malonyl-CoA from acetyl-CoA, which is a crucial step in fatty acid synthesis, and FAS converts malonyl CoA into palmitate, which is the first fatty acid product of de novo lipogenesis [25]. In the present study, we observed that AE significantly reduced the mRNA expression of PPARγ and FAS in the adipose tissue. Furthermore, the mRNA expression of C/EBPα, SREBP1-c, and ACC showed a tendency, though not significant, to decrease. AMPK regulates several metabolic reactions. AMPK-knockout mice fed a high-fat diet showed metabolic complications. In contrast, high-fat diet-fed mice with increased AMPK activation showed reduced metabolic dysfunction [26]. AMPK is known to suppress de novo lipogenesis and activate fatty acid oxidation. Phosphorylation of ACC and SREBP-1c by AMPK inhibits enzyme activity [27]. To analyze if the suppression of lipogenesis by AE was mediated through the AMPK pathway, we measured the levels of AMPK and ACC using western blotting. It was observed that AE significantly increased the levels of p-AMPK, which in turn resulted in the phosphorylation of ACC, indicating that the anti-obesity effect of AE is mediated through the AMPK pathway. Previous phytochemical analyses of *A. distichum* showed the presence of several compounds including acteoside, isoacteoside, rutin, chlorogenic acid, caffeic acid, taxifolin and ferulic acid [28]. Some of these compounds have been reported to have AMPK activation properties [29,30,31]. Taken together, it can be concluded that the anti-obesity effect of AE may be attributed to these compounds. Compounds from natural sources such as ginseng, licorice, and turmeric have shown anti-obesity effects by increased activation of AMPK and are gaining attention as potential anti-obesity agents [32,33,34]. The human effective concentration (equivalent to 300 mg/kg body weight) of AE is 24.3 mg/kg body weight, which might efficiently improve obesity in human subjects.

## 5. Conclusions

In summary, the results of our study demonstrated that AE significantly reduced HD-induced body weight gain, improved serum biochemical parameters, reduced ectopic lipid accumulation, and prevented adipose tissue dysfunction. In addition, AE suppressed lipogenesis in the adipose tissue by increasing AMPK and ACC phosphorylation. *A. distichum* has been reported to have health benefits such as anti-inflammatory, anti-diabetic, antioxidant, and anticancer effects [9,35,36,37]. In the present study, for the first time, we showed that *A. distichum* has anti-obesity effects, which are mediated through the AMPK pathway. It can be concluded that *A. distichum* is a potential candidate for the management of obesity and its effect on humans should be evaluated through clinical trials.

## Figures and Tables

**Table 1:** List of primers used for PCR.

Gene Name	Primers	Sequence (5′–3′)
*PPARγ*	Forward	GCCCACCAACTTCGGAATC
	Reverse	TGCGAGTGGTCTTCCATCAC
*C/EBPα*	Forward	GAGCTGAGTGAGGCTCTCATTCT
	Reverse	TGGGAGGCAGACGAAAAAAC
*SREBP1c*	Forward	CCAGAGGGTGAGCCTGACAA
	Reverse	AGCCTCTGCAATTTCCAGATCT
*FAS*	Forward	GAAGTGTCTGGACTGTGTCATTTTTAC
	Reverse	TTAATTGTGGGATCAGGAGAGCAT
*ACC*	Forward	GCCTCTTCCTGACAAACGAG
	Reverse	TAAGGACTGTGCCTGGAACC
*GAPDH*	Forward	FCATGGCCTTCCGTGTTCCTA
	Reverse	GCGGCACGRCAGATCCA

**Table 2:** Effect of AE on serum biochemical parameters in the experimental mice.

Serum Biomarkers	ND	HD	GE300	AE100	AE300
**ALT (U/L)**	44.9 ± 5.9 ^b^	196.8 ± 108.5 ^a^	74.9 ± 22.3 ^b^	55.2 ± 20.7 ^b^	41.4 ± 9.3 ^b^
**AST (U/L)**	56.9 ± 6.5 ^b^	114.4 ± 61.3 ^a^	86.3 ± 18.9 ^b^	71.6 ± 12.1 ^b^	69.6 ± 6.8 ^b^
**TC (mg/dL)**	127.0 ± 9.0 ^c^	220.0 ± 21.0 ^a^	168.0 ± 22.0 ^b^	176.0 ± 9.0 ^b^	162.0 ± 14.0 ^b^
**TG (mg/dL)**	126.0 ± 13.0 ^b^	247.0 ± 60.0 ^a^	118.0 ± 17.0 ^b^	123.0 ± 20.0 ^b^	124.0 ± 9.0 ^b^
**GLU (mg/dL)**	256.0 ± 39.1 ^c^	460.3 ± 69.6 ^a^	316.1 ± 38.1 ^bc^	320.0 ± 20.4 ^b^	303.5 ± 38.1 ^bc^
**HDL-C (mg/dL)**	98.8 ± 6.8 ^c^	159.8 ± 12.0 ^a^	138.1 ± 15.4 ^b^	140.9 ± 19.6 ^b^	142.6 ± 8.3 ^ab^
**LDL-C (mg/dL)**	14.5 ± 1.9 ^b^	35.2 ± 8.2 ^a^	17.3 ± 3.5 ^b^	20.2 ± 3.4 ^b^	18.1 ± 1.8 ^b^
**Leptin (pg/mL)**	225.2 ± 20.9 ^c^	484.6 ± 60.4 ^a^	421.3 ± 101.2 ^a^	433.5 ± 76.9 ^a^	318.6 ± 14.1 ^b^

Mice were fed a high-fat diet and orally administered GE (300 mg/kg b.w) or an AE (leaf extract) at low dose (100 mg/kg b.w) or high dose (300 mg/kg b.w) for 8 weeks. ALT, alanine aminotransferase, AST, aspartate aminotransferase, TC, total cholesterol, TG, triglycerides, GLU, glucose, HDL-C, high-density lipoprotein cholesterol, LDL-C, low-density lipoprotein-cholesterol. ND, normal diet, HD, high-fat diet, GE300, high-fat diet + Garcinia 300 mg/kg b.w, AE100, high-fat diet + 100 mg/kg b.w, AE300, high-fat diet + 300 mg/kg b.w. Values are expressed as mean ± SEM, *n* = 10. The varying superscript letters indicate a statistically significant difference among the groups by Duncan’s test of ANOVA, *p* < 0.05.
